# 10(E)-Pentadecenoic Acid Inhibits Melanogenesis Partly Through Suppressing the Intracellular MITF/Tyrosinase Axis

**DOI:** 10.3390/antiox13121547

**Published:** 2024-12-17

**Authors:** Min-Kyeong Lee, Kyoung Mi Moon, Su-Yeon Park, Jaeseong Seo, Ah-Reum Kim, Bonggi Lee

**Affiliations:** 1 Department of Food Science and Nutrition, Pukyong National University, Busan 48513, Republic of Korea; 3633234@hanmail.net (M.-K.L.); omkksm@pukyong.ac.kr (K.M.M.); syj5528@pukyong.ac.kr (J.S.); 2 Biotechnology Research Division, National Institute of Fisheries Science, Busan 46083, Republic of Korea; 3 Department of Smart Green Technology Engineering, Pukyong National University, Busan 48513, Republic of Korea; suzan@pukyong.ac.kr (S.-Y.P.); kimar0107@pukyong.ac.kr (A.-R.K.); 4 Marine Integrated Biomedical Technology Center, The National Key Research Institutes, Pukyong National University, Busan 48513, Republic of Korea

**Keywords:** 10(E)-pentadecenoic acid, melanogenesis, MITF, tyrosinase

## Abstract

Melanogenesis, the biological process responsible for melanin synthesis, plays a crucial role in determining skin and hair color, photoprotection, and serving as a biomarker in various diseases. While various factors regulate melanogenesis, the role of fatty acids in this process remains underexplored. This study investigated the anti-melanogenic properties of 10(E)-pentadecenoic acid (10E-PDA) through both in silico and in vitro analyses. SwissSimilarity was utilized to predict the functional properties of 10E-PDA by comparing it with structurally similar lipids known to exhibit anti-melanogenic effects. Subsequent in vitro experiments demonstrated that 10E-PDA significantly reduced melanin production and intracellular tyrosinase activity in α-MSH (melanocyte-stimulating hormone)-stimulated B16F10 melanoma cells without exhibiting significant cytotoxicity at concentrations up to 15 μM. Further mechanistic studies revealed that 10E-PDA inhibited the nuclear translocation of microphthalmia-associated transcription factor (MITF), consistent with the decrease observed in p-MITF protein levels. It also decreased the mRNA levels of tyrosinase-related proteins (TRP-1, TRP-2) and tyrosinase, while reducing the protein levels of TRP-1 and tyrosinase, but not TRP-2. These findings suggest that 10E-PDA exerts its anti-melanogenic effects by modulating the MITF/tyrosinase axis, presenting potential therapeutic implications for skin pigmentation disorders.

## 1. Introduction

Melanogenesis, the intricate biological process underlying the synthesis of melanin pigment, holds profound significance in both physiological and pathological contexts. From determining skin and hair coloration to influencing photoprotection and serving as a biomarker in various diseases, melanogenesis exemplifies the complexity and importance of cellular pigment regulation [1].

Melanogenesis is regulated by various factors, including the availability of precursors such as tyrosine and the activity of key enzymes like tyrosinase [1,2]. At its core, melanogenesis is orchestrated by a series of enzymatic reactions within specialized organelles called melanosomes, primarily located in melanocytes. Tyrosinase, the key enzyme in melanogenesis, catalyzes the conversion of tyrosine to dopaquinone, a pivotal step in melanin synthesis. This process is further modulated by a constellation of regulatory proteins, including tyrosinase, the key enzyme in melanogenesis, TRP-1 and TRP-2, MITF, and various signaling pathways [3,4,5]. Central to this regulatory network is the transcription factor MITF, which acts as a master regulator of melanocyte development, differentiation, and pigment production. MITF orchestrates the expression of key melanogenic enzymes and proteins, thus exerting precise control over melanin synthesis [3,4,5]. Furthermore, melanogenesis is intricately intertwined with cellular signaling pathways, including the cyclic adenosine 3′,5′-monophosphate (cAMP)/protein kinase A (PKA) pathway, the mitogen activated protein kinase (MAPK)/extracellular signal-regulated kinase (ERK) pathway, and the Wnt/β-catenin pathway, which modulate MITF activity and melanin production in response to various extracellular cues [3]. Understanding the molecular mechanisms underpinning melanogenesis is pivotal for unraveling the complexities of pigmentation biology and holds immense therapeutic potential. Modulating the melanogenesis pathway offers the potential to manage a variety of skin pigmentation disorders, such as vitiligo, melasma, and melanoma, and this modulation is critical to the development of new treatments.

Fatty acids play pivotal roles in cellular physiology, influencing diverse biological processes ranging from energy metabolism to cellular signaling. Beyond their traditional functions, emerging research has unveiled their intriguing involvement in modulating melanogenesis, the intricate process responsible for the production of melanin pigment. Fatty acids, classified by their chain length and degree of saturation, exhibit distinct effects on melanin synthesis. Saturated fatty acids, such as palmitic acid, with a short-chain structure of 16 carbons, are known to stabilize tyrosinase activity and promote melanosome maturation, leading to enhanced melanin production. In contrast, unsaturated fatty acids, including oleic acid, linoleic acid, linolenic acid, arachidonic acid (C20:4), and docosahexaenoic acid (C22:6), with medium to long chains of 18–22 carbons, have been shown to inhibit melanin synthesis. These unsaturated fatty acids promote tyrosinase degradation via the ubiquitin-proteasome system and may also disrupt melanogenesis-related signaling pathways, contributing to their anti-melanogenic effects [6,7]. While saturated and unsaturated fatty acids generally follow distinct trends in their effects on melanogenesis, exceptions like azelaic acid highlight the complexity of these interactions. Azelaic acid, a short-chain saturated nonanedioic acid, is widely recognized for its effective skin-lightening properties and is commonly used in dermal creams. Unlike typical saturated fatty acids, azelaic acid inhibits melanin production by acting as a competitive inhibitor of tyrosinase and disrupting melanocyte metabolism. This example underscores that factors beyond saturation, such as chain length and functional group placement, play a critical role in determining the impact of fatty acids on pigmentation [8].

Recent studies suggest that certain fatty acids regulate melanogenesis through direct interaction with key enzymes like tyrosinase and by modulating intracellular signaling pathways involved in melanin production [6]. These contrasting effects underscore the significance of fatty acid structure in pigmentation biology. Deciphering the complex interactions between fatty acids and melanin production is highly relevant across fields such as dermatology, cosmetology, and oncology [9]. Insights from such studies could pave the way for innovative therapeutic approaches to address skin pigmentation disorders, melanoma, and other conditions associated with melanin synthesis [6].

Among these, one fatty acid of emerging interest is 10(E)-pentadecenoic acid (10E-PDA), a monounsaturated fatty acid comprising 15 carbon atoms in its long chain [10]. Previous studies have employed 10E-PDA in exploring alternative β-oxidation pathways [11,12]. Despite its biochemical characterization decades ago [13], its role in melanogenesis remains poorly understood. Emerging evidence suggests that 10E-PDA may influence melanin synthesis, but the molecular mechanisms underlying this effect have not yet been determined. To address these knowledge gaps, we conducted both in silico and in vitro analyses to investigate the potential anti-melanogenic effects of 10E-PDA, focusing on its modulation of intracellular pathways and enzymatic activity related to melanin production.

## 2. Materials and Methods

### 2.1. Materials

2,2-diphenyl-1-picrylhydrazyl (DPPH), copper (II) chloride (CuCl_2_), neocuproine, 2′,7′-dichlorodihydrofluorescein diacetate (DCF-DA), α-melanocyte-stimulating hormone (α-MSH), L-DOPA, L-tyrosine, and tyrosinase from mushrooms were purchased from Sigma. 10E-PDA was dissolved in dimethyl sulfoxide (DMSO) to prepare the working solution.

### 2.2. DPPH Radical Scavenging Assay

The DPPH radical scavenging activity of 10E-PDA was measured using a previously published method [14]. Briefly, 5 μL of 10E-PDA at various concentrations (1–60 μM) and 245 μL of 0.1 mM DPPH solution dissolved in 80% methanol were mixed in a 96-well plate. After reacting in the dark for 30 min, absorbance was measured at 517 nm using a microplate reader (AMR-100, Allsheng, Hangzhou, China).

### 2.3. CUPRAC Method

CUPRAC analysis was performed with reference to previously published methods [15]. CUPRAC reagent was prepared by diluting a 10 mM CuCl_2_ solution and a 7.5 mM neocuproine solution with distilled water. Then, 2 μL each of various concentrations of 10E-PDA (1–60 μM) was dispensed into a 96-well plate and mixed with 198 μL of CUPRAC reagent. Afterwards, it was incubated at 37 °C for 10 min and the absorbance was measured at 450 nm. A standard curve was set up using ascorbic acid.

### 2.4. Mushroom Tyrosinase Activity Assay

We conducted a Mushroom Tyrosinase Activity Assay by referring to the experimental method described in a previously published paper [16]. 10E-PDA was dispensed into a 96-well plate with 50 mM sodium phosphate buffer (pH 6.5) containing 1 mM L-tyrosine and diluted with water to final concentrations of 1, 5, 15, and 60 µM. Before the addition of the enzyme, the absorbance at 490 nm of each well was measured after shaking for 10 seconds with a microplate reader. Subsequently, mushroom tyrosinase (1000 U/mL) was added to each well, and the reaction was conducted at 37 °C while preventing light exposure. The reaction was conducted at 37 °C for 20 min to ensure a linear correlation between absorbance and tyrosinase activity. The absorbance at 490 nm of each well was measured again after shaking for 10 s.

### 2.5. Cell Culture

The B16F10 murine melanoma cell line and Hs68 human dermal fibroblast cell line were purchased from the American Type Culture Collection (ATCC, Rockville, MD, USA). The human keratinocyte cell line HaCaT was kindly provided by the Korea Institute of Oriental Medicine (Daegu, Republic of Korea). All cells were cultured in Dulbecco’s modified Eagle’s medium (DMEM) supplemented with heat-inactivated fetal bovine serum (FBS) and 1% penicillin–streptomycin (P/S) in an environment of 5% CO_2_ at 37 °C.

### 2.6. Cell Viability

MTS assay (CellTiter96^®^ AQueous One Solution Cell Proliferation Assay Kit, Promega, Madison, WI, USA) was conducted to validate the cytotoxic effect of 10E-PDA. B16F10 murine melanoma cells (1 × 10^4^ cells/well), Hs68 human fibroblast cells (5 × 10^3^ cells/well), and HaCaT keratinocytes (5 × 10^3^ cells/well) were each seeded in 96-well plates. After incubating for 24 h, when the cells reached approximately 80% confluence, 10E-PDA was diluted to various concentrations (1–100 µM) in serum-free media, applied to the cells, and further incubated for 24 h. Following removal of the media, MTS solution was suspended in each well, followed by a 1 h incubation while avoiding exposure to light at 37 °C. Subsequently, the absorbance at 490 nm was measured in each well using a microplate reader.

### 2.7. Measurement of Melanin Contents

Melanin content was quantified and measured using a previously published method [17]. B16F10 murine melanoma cells were seeded in 6-well plates at a density of 5 × 10^3^ cells per well. After 24 h of incubation at 37 °C, 10E-PDA was diluted to various concentrations (1, 5, and 15 µM) in DMEM and then applied to the cells and incubated for 1 h. After 1 h of treatment with 10E-PDA, α-MSH (500 nM) was directly added to the same medium, and the cells were incubated at 37 °C for a further 6 days. Cells were then collected, lysed using 1N NaOH, and dissolved at 60 °C for 1 h. The absorbance of the dissolved cell lysate was measured at 490 nm, and the values were normalized by quantifying using the BCA method.

### 2.8. Intracellular Tyrosinase Activity Assay

Intracellular tyrosinase activity was measured as described in a previously published method [18]. B16F10 cells (5 × 10^4^ cells/well) were seeded and treated with 10E-PDA at concentrations of 1, 5, and 15 μM for 1 h. After 1 h of treatment with 10E-PDA, α-MSH (500 nM) was directly added to the same medium, and cells were incubated for 3 days. After the treatment period, cells were lysed using 50 mM sodium phosphate buffer (pH 6.5) containing 1% Triton X-100 and 0.1 mM phenylmethyl sulfonyl fluoride (PMSF). The lysates were centrifuged at 12,000× *g* for 30 min at 4 °C, and the resulting supernatant was collected. The supernatant was mixed with L-DOPA (2 mg/mL) and incubated at 37 °C for 1 h. Absorbance was measured at 490 nm to evaluate tyrosinase activity.

### 2.9. Real-Time PCR

Real-time PCR was conducted following a previously described protocol [19]. Briefly, RNA extraction from B16F10 melanoma cells was performed utilizing the RiboEX^TM^ reagent (GeneAll, Seoul, Republic of Korea). Subsequently, RNA was reverse transcribed into cDNA using the SmartGene Compact cDNA Synthesis Kit (SMART GENE, Daejeon, Republic of Korea). Quantitative real-time PCR analysis was conducted using the TOPreal^TM^ SYBR Green qPCR PreMIX (Enzynomics, Daejeon, Republic of Korea) and the QuantStudio^TM^ 1 Real-Time PCR system (Applied Biosystems, Foster City, CA, USA), following manufacturer’s instructions. Gene expression levels were assessed and determined using the 2^−ΔΔCT^ method [20]. The primer sequences used are available in Table 1.

### 2.10. Western Blotting

We conducted Western blot analysis following the method described in a previously published paper [21]. Proteins from B16F10 cells were extracted using the ExKine™ Total Protein Extraction Kit (KTP3006, Abbkine, Atlanta, GA, USA), and the supernatant was separated via centrifugation at 14,000× *g* and 4 °C for 15 min. The protein concentration of each supernatant was determined using the BCA assay and standardized to equal concentrations. Subsequently, proteins were separated via PAGE and transferred onto a PVDF membrane. The membranes were washed with Tris-buffered saline (TBS) containing 0.02% Tween 20 (TBST), followed by blocking for 1 h at room temperature with TBST containing 5% skim milk. Subsequently, following overnight incubation with the primary antibody at 4 °C, the membrane was washed and then incubated with the secondary antibody for 1 h and 30 min. After three additional washes with TBST for 5 min each, immunoreactivity was measured using an ECL detection kit. Densitometric analysis of the data was performed using a bioanalytical imaging system (Azure 300, Azure Biosystems, Dublin, CA, USA) and ImageJ software version 1.54f (NIH, Rockville, MD, USA). The primary antibodies employed in this study included TRP-1 (sc-25543), TRP-2 (sc-25544), tyrosinase (sc-7834), and β-actin (sc-47778, sc-81178), all obtained from Santa Cruz Biotechnology. The p-MITF antibody (PA5-104707) was sourced from Thermo Fisher Scientific (Waltham, MA, USA).

### 2.11. Immunofluorescence

Immunofluorescence staining was performed using a previously published method [22]. Briefly, B16F10 cells were fixed with 4% formalin and then treated with 0.5% Triton X-100 for 5 min. After blocking by treatment with blocking solution for 30 min, the cells were incubated with MITF antibody (1:100 dilution, sc-515925, Santa Cruz Biotechnology, Santa Cruz, CA, USA) overnight at 4 °C. After incubation, the primary antibody was removed and incubated with FSD™ 594 (1:50 dilution, RSA1145 BioActs, Incheon, Republic of Korea) for 1 h. Afterward, the nuclei of the cells were counterstained with DAPI, visualized using a fluorescence microscope (LS40, LE.AM Solution Inc., Siheung-si, Republic of Korea), and images were taken.

## 3. Results

### 3.1. Structural and Functional Analysis of 10E-PDA

**Figure 1:**
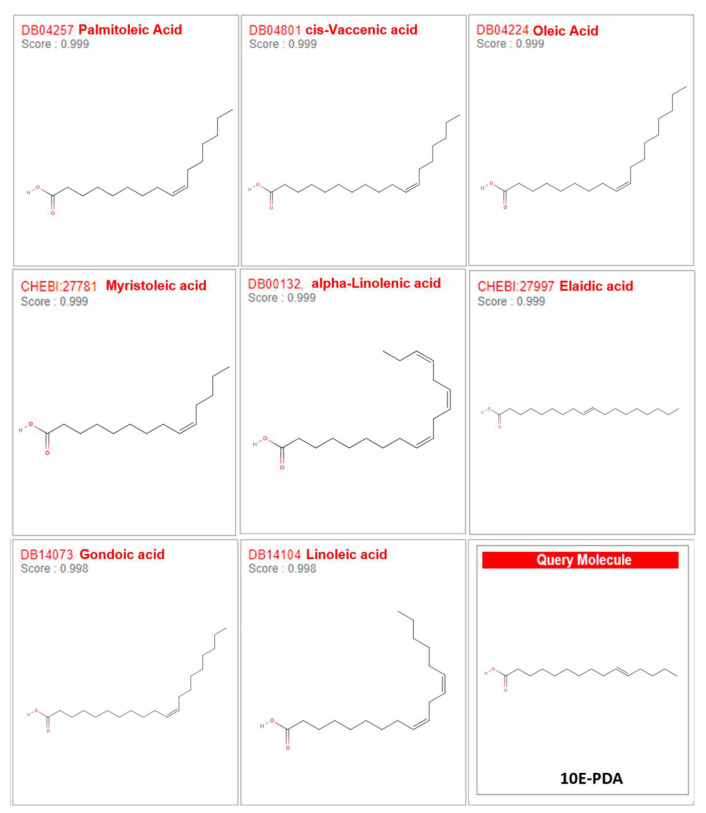
Similarity analysis of 10E-PDA with other compounds. SwissSimilarity was used to compare 10E-PDA with structurally similar compounds to predict its potential functions in the skin. 10E-PDA, which contains a trans double bond between the 10th and 11th carbons, exhibited very close similarity to compounds such as palmitoleic acid, cis-vaccenic acid, oleic acid, myristoleic acid, α-linolenic acid, elaidic acid, gondoic acid, and linoleic acid, with similarity scores ranging from 0.998 to 0.999. Among these, several compounds, including palmitoleic acid, α-linolenic acid, and linoleic acid, are known to have anti-melanogenic properties.

10E-PDA is a monounsaturated fatty acid with a 15-carbon chain that includes a carboxyl group at one end and a single trans double bond located between the 10th and 11th carbons from the carboxyl end [10]. SwissSimilarity, an advanced web-based tool, allows for the rapid screening of large-scale libraries, encompassing drugs, bioactive small molecules, commercially available compounds, and an extensive array of virtual compounds that are easily synthesized from commercially available reagents [23,24]. This tool efficiently identifies potential bioactive compounds using molecular fingerprints and both superpositional and non-superpositional 3D shape similarity methods. These approaches facilitate the quick comparison and identification of similar compounds, thereby aiding in drug discovery, molecular mechanism research, and lead compound optimization [23]. Given that the functions of 10E-PDA in skin aging remain unexplored, we employed SwissSimilarity to compare it with structurally similar lipids and predict its potential functions in the skin. The results showed that 10E-PDA exhibited very close similarity to palmitoleic acid (similarity score 0.999), cis-vaccenic acid (similarity score 0.999), oleic acid, myristoleic acid (similarity score 0.999), α-linolenic acid (similarity score 0.999), elaidic acid (similarity score 0.999), gondoic acid (similarity score 0.998), and linoleic acid (similarity score 0.998) (Figure 1). Of these lipids, palmitoleic acid [25], α-linolenic acid [26], and linoleic acid [26] exhibited anti-melanogenic properties (Figure 1). Given the structural similarity of 10E-PDA to these lipids, we hypothesized that its trans double bond configuration might also inhibit melanogenesis. To test this hypothesis, we conducted a series of in vitro and cell-free experiments to evaluate the potential anti-melanogenic effects of 10E-PDA.

### 3.2. 10E-PDA Suppresses Intracellular Tyrosinase Activity and Melanin Levels in B16F10 Cells

**Figure 2:**
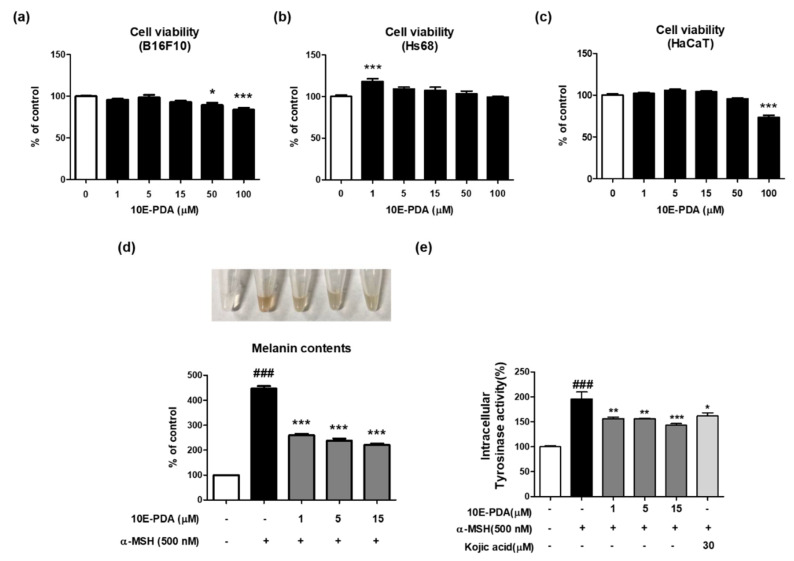
Effects of 10E-PDA on inhibiting melanogenesis in α-MSH-stimulated B16F10 melanoma cells. (**a**) B16F10, (**b**) HS68, and (**c**) HaCaT cells were treated with increasing concentrations of 10E-PDA (1–100 μM) for 24 h, after which cell viability was assessed (n = 4 per group). (**d**,**e**) B16F10 cells were pretreated with different concentrations of 10E-PDA (1–15 μM) or kojic acid (30 μM) for 1 h, followed by exposure to α-MSH (500 nM) for 6 days to measure melanin content (**d**) or for 3 days to examine tyrosinase activity (**e**). In both panels, the white bar represents the untreated control group without α-MSH stimulation, the black bar represents the α-MSH-stimulated control group, and the dark gray bars indicate the 10E-PDA-treated groups under α-MSH stimulation. In panel (**e**), the light gray bar represents the positive control group treated with kojic acid under α-MSH stimulation. Data are shown as mean ± SEM. ^###^ *p* < 0.001 compared with the untreated control group, and * *p* < 0.05, ** *p* < 0.01, *** *p* < 0.001 compared with the α-MSH-treated group.

To evaluate the safety of 10E-PDA, cytotoxicity assays were performed using various skin cell lines: B16F10 (mouse melanoma), Hs68 (human fibroblasts), and HaCaT (human keratinocytes). As a result, 10E-PDA did not show significant cytotoxicity in Hs68 cells (Figure 2a) up to 100 μM, but showed significant cytotoxicity in B16F10 (Figure 2b) and HaCaT (Figure 2c) cells at 50 μM and 100 μM, respectively. Therefore, 10E-PDA was used in subsequent experiments at a concentration range of 1 to 15 μM.

To analyze cellular melanin production, B16F10 melanoma cells were pretreated with 10E-PDA (1–15 µM) for 1 h, then treated with α-MSH and cultured for 6 days to induce melanin accumulation. α-MSH treatment significantly upregulated cellular melanin content by approximately 400% compared to the control group, and 10E-PDA treatment reduced this increase in a dose-dependent manner (Figure 2d). Based on these results, we assessed intracellular tyrosinase activity to explore the potential mechanism behind the anti-melanogenic effect of 10E-PDA. 10E-PDA treatment decreased intracellular tyrosinase activity in a concentration-dependent manner after α-MSH treatment (Figure 2e).

### 3.3. 10E-PDA Exhibits Antioxidant Activity but May Not Directly Inhibit Tyrosinase Activity

**Figure 3:**
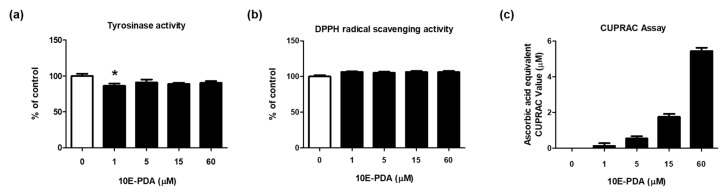
Cell-free tyrosinase and antioxidant activities of 10E-PDA. (**a**) Tyrosinase activity of 10E-PDA was measured using a mushroom tyrosinase activity assay. (**b**) The antioxidant capacity of 10E-PDA was evaluated using DPPH radical scavenging activity. (**c**) The copper ion reduction capacity of 10E-PDA was determined using CUPRAC analysis, with results compared with the reference ascorbic acid. In all panels, the white bar represents the untreated control group, and the black bars indicate the 10E-PDA-treated groups at various concentrations (1–60 μM). Data are presented as mean ± standard error (n = 3). * *p* < 0.05 compared with the untreated control group.

To assess the direct effect of 10E-PDA on tyrosinase activity, we measured its tyrosinase inhibitory activity in a cell-free system. Treatment with 1–60 µM 10E-PDA slightly inhibited tyrosinase activity at 1 µM, but did not exhibit a significant inhibitory effect at higher concentrations (Figure 3a). These results suggest that 10E-PDA may not have a strong effect on tyrosinase activity.

Antioxidants such as glutathione and ascorbic acid are known to interrupt the chain reaction and impede melanin formation by reacting with intermediate products [27]. Therefore, we evaluated the antioxidant activity of 10E-PDA using the DPPH radical scavenging activity assay and the CUPRAC assay. 10E-PDA treatment significantly increased the reducing power in the CUPRAC assay, but radical scavenging activity was not observed in the DPPH assay (Figure 3b,c).

### 3.4. 10E-PDA Inhibits MITF Translocation into the Nucleus and Decreases the mRNA and Protein Expression Levels of TRP-1, TRP-2, and Tyrosinase in α-MSH-Stimulated B16F10 Cells

**Figure 4:**
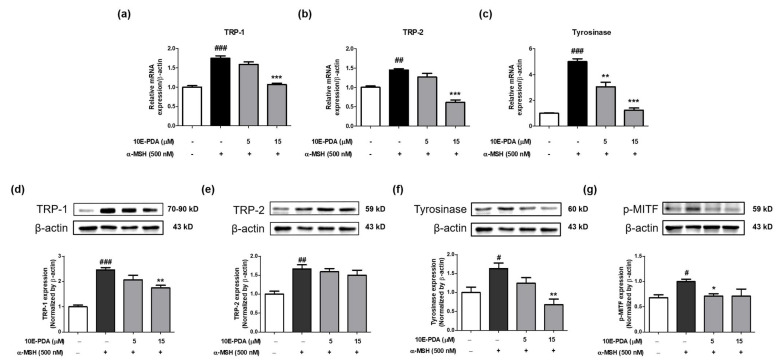
Impact of 10E-PDA on gene and protein expression of melanogenesis-related targets. To analyze the effect of 10E-PDA on melanogenesis markers, before stimulation with α-MSH (500 nM) over six days, B16F10 melanoma cells were pre-treated with 10E-PDA at concentrations of 5 and 15 μM for one hour. Quantitative PCR (qPCR) was employed to measure mRNA expression levels for (**a**) TRP-1, (**b**) TRP-2, and (**c**) tyrosinase. Additionally, Western blot analysis was conducted to evaluate protein levels of (**d**) TRP-1, (**e**) TRP-2, (**f**) tyrosinase, and (**g**) p-MITF across groups (n = 3/group). In all panels, the white bar represents the untreated control group (no α-MSH stimulation), the black bar represents the α-MSH-stimulated control group, and the dark gray bars indicate the 10E-PDA-treated groups (α-MSH-stimulated). Results are presented as mean ± SEM. ^#^ *p* < 0.05, ^##^ *p* < 0.01 and ^###^ *p* < 0.001 compared with the non-treated control group, and * *p* < 0.05, ** *p* < 0.01 and *** *p* < 0.001 compared with the α-MSH-treated group.

**Figure 5:**
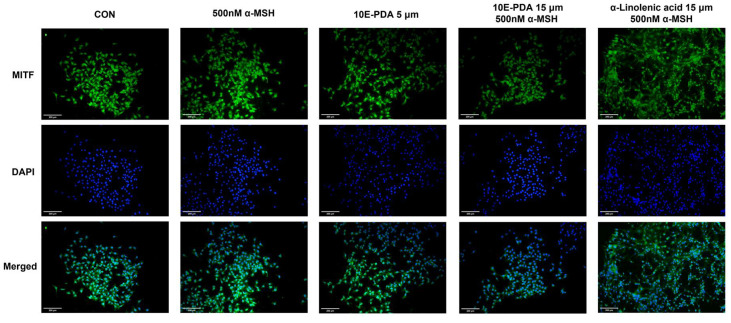
Effects of 10E-PDA on MITF translocation. Immunofluorescence analysis was performed to evaluate the inhibitory effect of 10E-PDA on the nuclear translocation of MITF. B16F10 cells were treated with 10E-PDA (5 and 15 μM) and linolenic acid (15 μM), and fluorescence microscopy evaluation was performed. MITF was detected using anti-MITF monoclonal antibody and then detected using FSD^TM^-conjugated secondary antibody. This analysis revealed the localization of MITF, thereby elucidating the effect of 10E-PDA on MITF nuclear translocation (Scale bars represent 200 μm).

To elucidate the molecular mechanism of anti-melanogenesis mediated by 10E-PDA, we investigated the expression levels of mRNA and proteins related to melanogenesis. α-MSH significantly increased the mRNA and protein expression of TRP-1, TRP-2, and tyrosinase, whereas 10E-PDA treatment significantly decreased the mRNA levels of these genes. Western blot analysis showed that 10E-PDA decreased the protein expression of TRP-1, tyrosinase, and p-MITF, but did not affect TRP-2 (Figure 4a–f). These results may be due to various posttranscriptional regulatory mechanisms and protein stability factors, which may explain the observed discrepancies [28,29]. Given that MITF is a key transcription factor regulating TRP-1, TRP-2, and tyrosinase, we used immunofluorescence analysis to evaluate the effect of 10E-PDA on MITF nuclear translocation. Fluorescence microscopy showed that 10E-PDA treatment inhibited α-MSH-induced nuclear translocation of MITF in B16F10 melanoma cells, with a similar inhibitory effect at the same concentration as α-linolenic acid, a fatty acid known for its whitening effect (Figure 5). These results suggest that 10E-PDA inhibits cellular melanogenesis by preventing MITF from translocating to the nucleus and reducing the expression of TRP-1, TRP-2, and tyrosinase.

## 4. Discussion

The results of this study provide compelling evidence for the anti-melanogenic properties of 10E-PDA, a monounsaturated fatty acid capable of regulating melanogenesis by targeting key elements such as MITF and tyrosinase. By inhibiting melanin production, 10E-PDA emerges as a promising candidate for therapeutic applications in managing hyperpigmentation disorders.

Melanogenesis is a highly regulated process involving a network of enzymes and regulatory proteins. The central role of MITF in this process, regulating the expression of key enzymes like tyrosinase, TRP-1, and TRP-2, makes it a crucial target for anti-melanogenic interventions [30]. The phosphorylation of MITF influences its activity and subcellular localization, thereby modulating melanin production. Our study suggests that 10E-PDA can inhibit melanogenesis in B16F10 melanoma cells by blocking MITF nuclear translocation and reducing downstream melanogenesis factors such as tyrosinase and TRP-1 at both the mRNA and protein levels. However, TRP-2 mRNA levels were reduced while protein levels remained unchanged, suggesting potential post-transcriptional regulation. This finding highlights the potential of 10E-PDA to be developed into a targeted therapeutic agent for skin pigmentation disorders.

In this study, α-linolenic acid, a well-known anti-melanogenic omega-3 fatty acid [7,26], served as a positive control. Similarly to 10E-PDA, α-linolenic acid effectively reduced melanin production, further validating the anti-melanogenic effects of 10E-PDA. This similarity in their effects can be explained by the structural resemblance between 10E-PDA and other anti-melanogenic lipids, such as α-linolenic acid [26], palmitoleic acid [25], and linoleic acid [26]. These structural similarities suggest the possibility of shared pathways in melanogenesis inhibition. Future studies comparing 10E-PDA with cis-unsaturated fatty acids like myristoleic acid and palmitoleic acid could clarify the impact of structural differences, particularly cis and trans double bond configurations, on melanogenesis. The use of SwissSimilarity for structural and functional predictions underscores the value of bioinformatics tools in identifying and validating potential bioactive compounds [31]. The structural similarity findings reinforce the likelihood that 10E-PDA and α-linolenic acid exert their effects through similar mechanisms, making 10E-PDA a promising candidate for further exploration. Notably, tyrosinase activity inhibition at 1 µM showed a slight deviation from the trend observed at other concentrations. While this result might appear unusual, it was consistently observed across replicates under our specific experimental conditions. This could indicate a potential concentration-specific interaction or previously unrecognized dynamics in enzyme-inhibitor interactions at low concentrations. However, further studies are required to validate and fully understand this observation. Until such studies are conducted, we suggest interpreting this finding cautiously as an initial observation.

Regarding the CUPRAC assay, while cupric salts might interfere with the testing of unsaturated fatty acids, the results for 10E-PDA were consistent and reproducible across all replicates under the specified conditions. This suggests that potential interference by cupric salts did not significantly affect the observed antioxidant activity. Nevertheless, the CUPRAC results should be interpreted with caution, as interference could vary depending on assay conditions and the specific properties of other test compounds. For the DPPH assay, the absence of radical scavenging activity by 10E-PDA aligns with previous findings for certain fatty acids and their derivatives. This likely reflects the chemical nature of 10E-PDA, which may not effectively interact with DPPH radicals due to differences in their mechanistic pathways. These observations highlight the limitations of these assays for certain compound classes, emphasizing the importance of using complementary methods to evaluate antioxidant activity comprehensively.

Our results show that 10E-PDA exhibited selective antioxidant activity in the CUPRAC assay and significantly reduced intracellular tyrosinase activity and melanin content in α-MSH-stimulated B16F10 cells, similarly to kojic acid. This reduction in tyrosinase activity and melanin content was associated with decreased MITF phosphorylation, thereby inhibiting its nuclear translocation and reducing the expression of melanogenesis-related enzymes such as tyrosinase [32]. Western blot analysis further indicated that p-MITF, rather than total MITF, plays a critical role in mediating the effects of 10E-PDA on melanogenesis. Total MITF levels showed no significant change, but pMITF levels were significantly reduced, directly impacting MITF translocation and tyrosinase expression. These findings underscore the importance of post-translational modifications of MITF in the anti-melanogenic effects of 10E-PDA.

While this study highlights the anti-melanogenic potential of 10E-PDA, further investigation is warranted to fully elucidate its precise mechanism of action. An important focus for future research could be the MAPK/ERK signaling pathway, which regulates MITF activity in response to oxidative stress [33]. It is plausible that 10E-PDA interacts with this pathway to modulate MITF phosphorylation, thereby influencing melanogenesis. Additionally, the ubiquitin–proteasomal system (UPS), which regulates the degradation of melanogenesis-related proteins such as tyrosinase through post-translational modifications like ubiquitination, may also play a role [34,35]. Investigating the effects of 10E-PDA on these pathways could provide novel insights into its anti-melanogenic properties and expand our understanding of its mechanism of action.

Finally, 10E-PDA holds significant potential for applications in the cosmetic field, offering multiple benefits. Fatty acids and their derivatives are vital components in cosmetic formulations due to their diverse functions, such as enhancing stability, performance, and product aesthetics [36]. They also have moisturizing and emollient properties that support the skin’s lipid barrier, reduce water loss, and promote hydration—essential for anti-aging and sensitive skin care [36,37,38]. In addition, fatty acids strengthen the skin’s protective barrier against environmental factors like UV radiation and pollutants [37]. Fatty acid derivatives, such as esters, are particularly valuable in emulsions like water-in-oil (W/O) and oil-in-water (O/W) systems, offering a range of physical and aesthetic benefits in cosmetic products.

## 5. Conclusions

This study revealed that 10E-PDA can inhibit melanogenesis by partially suppressing the intracellular MITF/tyrosinase axis. While further research is required to understand the detailed mechanisms and assess toxicity, our results indicate that 10E-PDA shows potential as a depigmenting and cosmeceutical agent.

## Figures and Tables

**Table 1:** Primer details for target gene amplification. The table summarizes the sequences of forward and reverse primers used for the amplification of TRP-1, TRP-2, and Tyrosinase genes. Gene-specific primers were designed based on NCBI Gene IDs to ensure accurate amplification for downstream molecular analyses.

Genes	NCBI Gene ID	Sequence (5′ → 3′)	
TRP-1	22178	Forward	GCT GCA GGA GCC TTC TTT CTC
		Reverse	GTC ATC AGT GCA GAC ATC GC
TRP-2	104042	Forward	CTC AGA GCTCGG GCT CAG TT
		Reverse	TGT TCA GCA CGC CAT CCA
Tyrosinase	22173	Forward	TTC TGC CTT GGC ACA GAC TT
		Reverse	GCA AGC TGT GGT AGT CGT CT

## Data Availability

Data are contained within this article.
